# Predicting current and future climate suitability for arecanut (*Areca catechu* L.) in India using ensemble model

**DOI:** 10.1016/j.heliyon.2024.e26382

**Published:** 2024-02-16

**Authors:** K.B. Hebbar, Abhin Sukumar P, Sanjo Jose V, Ramesh S V, Ravi Bhat

**Affiliations:** a Indian Council of Agricultural Research – Central Plantation Crops Research Institute, Kasaragod, Kerala, India; b Forest Research Institute, Dehradun, India

**Keywords:** Crop adaptability, Climate change, Ensemble projection, Habitat prediction, Species distribution, Modeling

## Abstract

Climate change has the potential to influence plant development, physiology, and distribution. Arecanut (*Areca catechu* L.), with its long life span of 60–70 years, thrives in a tropical habitat remains exposed to various abiotic and biotic factors. It is pertinent to comprehend the adaptation strategies of this crop towards climate change over time. The Biomod2 ensemble platform for species distribution modeling was utilized to predict the potential impact of climate change on the adaptability of the crop. The extracted study region of India was used for prediction, and the final run of 6 models ensemble includes 894 occurrence points and 9 climate variables with 80%–20% of training and validation sets. The model's outputs had area under curve (AUC) values of 0.943 and true skills statistics (TSS) of 0.741, which are regarded as accurate. The research area was categorized into five groups: very high, high, moderate, low, and very low. The examination involved assessing the shift in each category from the present to two prospective scenarios (shared socio-economic pathways; SSP 2–4.5 and SSP 5–8.5) projected for the 2050s and 2070s. A shift in the climate suitability area from ‘very high’ and ‘high’ categories to ‘moderate’ or ‘very low’ categories was observed suggesting the need for adaptive strategies to sustain the current yield levels. Amongst the regions, Karnataka state, which at present has more than 50% area under cultivation, is highly vulnerable and more area is coming under ‘very low’ and ‘low’ categories from eastern side. Meanwhile, in north eastern part of the country a shift in high suitable region from northwest to southwest is observed. Overall, the model prediction suggests that some parts of west and south interior regions of the country warrant immediate consideration in order to adapt to future climate change, whereas some part of north east can be considered for future cultivation.

## Highlights

• Climate suitability of arecanut in India predicted using ensemble platform Biomod2.
• Arecanut plantations in Karnataka is restricted by precipitation and temperature.
• In Northeast diurnal temperature variations are a major limitation.
• Phenotyping and genotyping modalities in arecanut against abiotic stress warranted.

## 1 Introduction

Arecanut (*Areca catechu* L.), is one of the economically important species of India belonging to family Arecaceae [1]. The palm is also cultivated in several other South Asian and South East Asian Countries such as Indonesia, Myanmar, China, Bangladesh, Thailand, Malaysia, Vietnam, Philippines, etc. [2]. It is a slender, tall, oriental palm with an economic life span of around 45–70 years. Under well managed conditions the palm flowers at around four years otherwise it requires six to seven years. The palm reaches its full bearing potential in about ten to fifteen years. Fruits require nine to twelve months to mature, that remains green when young and turns orange yellow or scarlet red upon ripening [3]. The seed or endosperm is used for chewing or masticatory purpose and possesses various medicinal properties [4].

Arecanut's traditional area of cultivation in India, during 1950s, was confined to the moderate elevations of western and eastern slopes of Western Ghats but thrives well at low altitudes [3]. However, the crop has gradually replaced other traditional crops spreading to high and low altitudes plains of south India as the palm fetches high economic returns. The area under arecanut in 2017-18 (0.505 m ha) had witnessed an increase to 0.704 and 0.731 during the years 2018-19 and 2019-20, respectively illustrating its rapid spread in India. In Karnataka, a state of India, alone the area under the palm had almost doubled from 0.28 to 0.48 m ha in the non-traditional areas during the same period. Currently, Karnataka occupies 52% of the total area of arecanut cultivation in India followed by Kerala, Assam, Tamil Nadu, West Bengal (http://www.dasd.gov.in).

While arecanut is capable of thriving in diverse climatic conditions, its productivity is significantly impacted by weather factors such as rainfall, relative humidity, and temperature [5,6]. According to Jose et al. [7], the yield of arecanut is primarily influenced by maximum temperature (T_max_), minimum temperature (T_min_), rainfall, and relative humidity (RH), accounting for more than 97% of the variation. Arecanut palms flourish in areas with substantial rainfall, typically 500 cm or more. However, they can also survive with as little as 75–150 cm of rainfall if the deficit is compensated by extensive irrigation during the summer months. In India, areca nut is grown in high rainfall areas such as Malnad of Karnataka (≥450 cm) and in low rainfall areas like plains of Karnataka or parts of Coimbatore district in Tamil Nadu (75 cm). Higher rainfall during nut development stage (June to July) significantly reduces the yield. In arecanut plantations the T_max_ should not exceed 36 °C, but a continuous temperature of 16 to 30 °C is preferable. In India, arecanut experiences T_min_ of 5 °C (North east, West Bengal) and T_max_ 40 °C (Vittal in Karnataka, Kannara in Kerala and eastern part) for few days in a year. The palms are unable to withstand extremes of temperatures and wide diurnal variations [8].

Prediction Models have projected that temperature in arecanut growing regions is anticipated to increase to an extent of 1.1 °C–2.6 °C as we approach the conclusion of the 21st century, and thus subjugating the crop to imminent threat of climate change [IPCC]. During the last decade alone, an increase of +0.4 °C in T_max_ and T_min_ was observed in Karnataka [9]. Climate suitability of perennial plantations like coconut in India [10] and rubber in Western Ghats and North East regions of India [11] is already predicted using MaxEnt model. Hence, predicting the climate suitability of arecanut will help in identifying suitable varieties or formulating appropriate management strategies in advance.

Anthropogenic climate change is expected to result in both the expansion and contraction of ecological ranges, posing a potential threat to a considerable number of species in the process [[12], [13], [14], [15]]. Species distribution models (SDMs), are valuable tools for determining the present and probable future geographic distributions [16,17]. Prediction of future climatic suitability of cash crops namely cocoa [18], jute [19], tea [20], and coconut [10] among others have opened up research on adaptive measures and sustainable cultivation of these crops. In order to predict species distributions based on environmental factors, a variety of SDM tools have been developed [[21], [22], [23]]. Nevertheless, owing to the multiple concepts and algorithms, each model possesses its own set of advantages and limitations, and if the input data are modified, performance of each model becomes uneven [21]. Biomod, a modelling platform built on R software, was created in 2003 to increase the precision of prediction, and has since gained widespread attention [24,25]. It encompasses various the species distribution modeling techniques including Artificial Neural Networks (ANNs), Classification Tree Analysis (CTA), Flexible Discriminant Analysis (FDA), Generalized Additive Models (GAMs), Generalized Boosted Models (GBMs), Generalized Linear Models (GLMs), MaxEnt, Multivariate Adaptive Regression Splines (MARS), Random Forest (RF) and Surface Range Envelope (SRE) Modeling. The initial conditions and model parameters could be customized to obtain precise results [26]. Therefore, this study used ensemble SDM, Biomod2, to analyze the drivers of current and future climate vulnerability of arecanut in India and to suggest suitable adaptation measures.

## 2 Materials and methods

### 2.1 Study area

The cultivation of arecanut palms in India occurs across diverse climatic and soil conditions, primarily within latitudes ranging from 8°4′ N to 20° N. Table 1 presents information on the major areca nut growing states in India, including details on the area cultivated at the district level, as well as climate and soil characteristics. Southwest region of the country is the traditional belt of arecanut cultivation in India. During 1950s, of the total 0.26 m ha area in India, 57% was in Kerala, 26% in Karnataka and 10% in Assam [3]. After 70 years Karnataka has 68% of the total area whereas Kerala's share is only 13% while in Assam the area remains unchanged. Area under Kerala is reduced mainly because of a disease called as Yellow leaf disease [27]. In Karnataka, on the other hand, the crop is spreading to non-traditional areas irrespective of the availability of suitable climate.

**Table 1:** The characteristic features of select key arecanut-growing districts in India, which served as input for Ensemble modeling, include information on humidity levels during the summer months (March, April, and May).

State	Area (000 ha)	Major districts	Latitude	Longitude	Soil type	Temperature range (°C)		Humidity (%)	Precipitation (mm)
						Minimum	Maximum	(Range)	
Karnataka	500.52	Chikmagalur	13.3161° N	75.7720° E	Clay loam	11.8–18.3	28.0–36.0	33–93	2019
		Shivamoga	13.9299° N	75.5681° E	Brown clay loamy, Red & Sandy	13.9–30.0	22.0–36.6	55–69	1813
		Davanagere	14.4644° N	75.9218° E	Red sandy & black	17.6–23.2	30.5–34.2	38–62	800
		Dakshina Kannada	12.8438° N	75.2479° E	Laterite & sandy loam	13.9–23.2	28–35.2	36–93	4089
		Tumkur	13.3379° N	77.1173° E	Red loamy, & black	15.0–17.4	31.0–37.1	11–90	554
		Chitradurga	14.2251° N	76.3980° E	Red sandy loam	15.7–17.8	33.9–38.0	11–90	508
		Uttara Kannada	14.7937° N	74.6869° E	Loamy laterite	13.0–25.0	25.0–33.0	40–80	2500
Kerala	96.92	Malappuram	11.0510° N	76.0711° E	Loamy	18.0–24.2	23.7–36.0	44–94	2877
		Kasaragod	12.4996° N	74.9869° E	Red sandy loam sandy	20.2–23.8	31.5–36.0	36–93	4245
		Kozhikode	11.2588° N	75.7804° E	Alluvial, lateritic	16.9–24.0	28.2–36.0	46–92	3592
		Wayanad	11.6287° N	76.0813° E	Laterite	23.0–26.0	33.0–35.0	55–73	2006
		Kannur	11.8745° N	75.3704° E	Sandy loam to clay	18.1–23.8	30.0–36.0	45–95	3831
		Palakkad	10.7867° N	76.6548° E	Black cotton soil	19.0–24.0	32.0–36.0	52–68	497
Assam	67.02	Sonitpur	26.6739° N	92.8577° E	Alluvial & sandy loam	11.0–25.0	24.0–33.0	64–80	1496
		Nagaon	26.3480° N	92.6838° E	Red & red loam	11.0–25.0	24.0–33.0	64–80	1498
		Golaghat	26.5239° N	93.9623° E	Inceptisol & Entisol	9.0–25.0	25.0–33.0	63–83	1551

### 2.2 Occurrence data collection

**Fig. 1:**
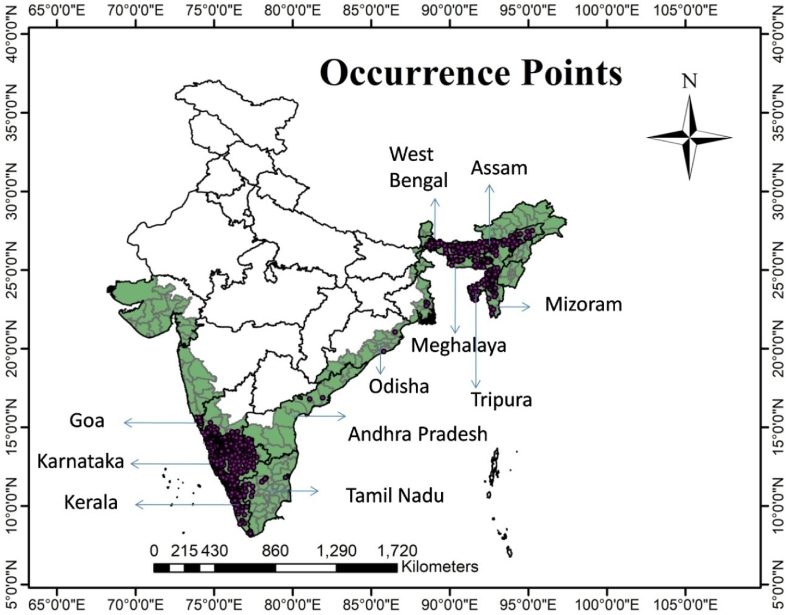
Study area and occurrence points of *Areca catechu* L. The green area represents the study area and the purple point indicates the arecanut occurrence points used in model running.

The information regarding the primary areca nut growing states and districts was obtained from the Directorate of Areca nut and Spices Development (DASD) website, accessible at https://www.dasd.gov.in. Distinct districts with substantial areca nut cultivation were chosen for inclusion in the study. For each of these selected districts, details such as the names and areas of villages with extensive areca nut cultivation were gathered. The corresponding coordinates (latitude x longitude) for these locations were determined using Google Earth maps, as detailed in a prior study [10]. These data, in conjunction with the point data acquired through the Global Positioning System (GPS) in earlier research, constituted the set of occurrence points. Spatial thinning at 5 km^2^ of total arecanut points performed using SDM toolbox and the remaining 894 points were used for the final model run (Fig. 1).

### 2.3 Bioclimatic variables selection

Nineteen distinct bioclimatic variables, as listed in Hebbar et al. [10], were obtained with a 2.5-min spatial resolution for both current (1970–2000) and future climate data. These data were retrieved from the WorldClim dataset version 2.1, accessible at https://www.worldclim.org, as of January 6, 2022. BCC-CSM2-MR, a climate model developed by Beijing Climate Center [28], was used to project the probable appropriate habitats of areca for two shared socioeconomic pathway scenarios (SSP 2–4.5 and SSP 5–8.5) for the periods, namely mid-term 2050 (2041–2060), and long-term 2070 (2061–2080). SSP 2–4.5 represents an intermediate development pathway, characterized by a radiation forcing of approximately 4.5 W/m2 in 2100. In contrast, SSP 5–8.5 signifies the high end of the spectrum for future pathways, featuring a radiation forcing of 8.5 W/m2 in 2100. To identify a specific set of variables that made substantial contributions to the models, Pearson correlation analysis was employed using the SDM Toolbox 2.0. This analysis involved the removal of one variable from each pair exhibiting correlations higher than 0.80 (Supplementary Table 1). Ten climate variables that exhibited high correlations were excluded, and the remaining nine bioclimatic variables were chosen for the model calibration (refer to Supplementary Table 1).Nine climate variables selected are bio1 (Annual Mean Temperature), bio 2 (Mean Diurnal Range (Mean of monthly (max temp - min temp)), bio 3 (Isothermality (bio 2/bio 7) ( × 100)), bio 7 (Temperature Annual Range (bio 5-bio 6)), bio12 (Annual Precipitation), bio14 (Precipitation of Driest Month), bio15 (Precipitation Seasonality (Coefficient of Variation)), bio18 (Precipitation of Warmest Quarter), bio19 (Precipitation of Coldest Quarter) for the ensemble model running.

### 2.4 Model

The biomod2 package, integrated into the R statistical program (version 4.2.0; R Development Core Team 2021, accessed in May 2022), was employed for running an ensemble model to determine the present and future distributions of Areca catechu L. The “biomod2” package supports ten model algorithms, including GLMs [29], generalized boosted models (GBMs) [30], generalized additive models (GAMs) [29], classification tree analysis (CTA) [31], artificial neural networks (ANNs) [32], surface range envelope (SRE) modeling [33], flexible discriminant analysis (FDA) [34], multivariate adaptive regression splines (MARS) [35], random forest (RF) [36], and MaxEnt [37].

### 2.5 Model calibration and validation

In the modeling process, 80% of the 894 samples from arecanut distribution data were chosen randomly to serve as training data, while 20% of the samples served as testing data. Three sets of pseudosampling points, each comprising 500 points randomly distributed, were generated. These pseudosampling points, along with the arecanut occurrence data, were utilized to create three sets of model inputs [38]. These sets were then incorporated into the model creation process three times, aiming to closely replicate the actual distribution and reduce spatial variation. Consequently, a total of 90 diverse models were developed and evaluated, involving three sets of sampling data, each processed through 10 individual modeling techniques, and repeated three times.

The performance of the models was assessed using the receiver operating characteristic (ROC) area under the curve (AUC) and true skill statistics (TSS) [39,40]. AUC values range from 0 to 1, where values between 0.5 and 0.7 signify poor model performance, between 0.7 and 0.9 indicate good performance, and values above 0.9 indicate excellent performance [41]. Similarly, TSS scores exceeding 0.6 are deemed acceptable [39,42]. For this study, individual models with AUC values greater than 0.875 and TSS values exceeding 0.625 were chosen for ensemble modeling.

### 2.6 Ensemble model (EM) for areca prediction

The ensemble model was employed to mitigate the uncertainty arising from various modeling algorithms and sample datasets [43,44]. First, SRE CTA, FDA, and GLM were excluded because these methods had average AUC and TSS value of less than 0.875 & 0.625 to generate the final ensemble models. Therefore the final ensemble had six algorithms, and a total of 54 various models (three sets of sampling data × 6 single modelling techniques × 3 repeats) were developed and evaluated. The results were used for the final analysis.

For mapping and calculating the various climate-suitable area classifications, we used weighted mean approach. The suitability maps generated by the model for both present and future scenarios ranged from 0 to 1000. These maps were reclassified into five suitability classes, namely Very Low (0–200), Low (200–400), Moderate (400–600), High (600–800), and Very High (800–1000), facilitating the analysis of climatic suitability from the current to future conditions.

## 3 Results

### 3.1 Model evaluation

**Fig. 2:**
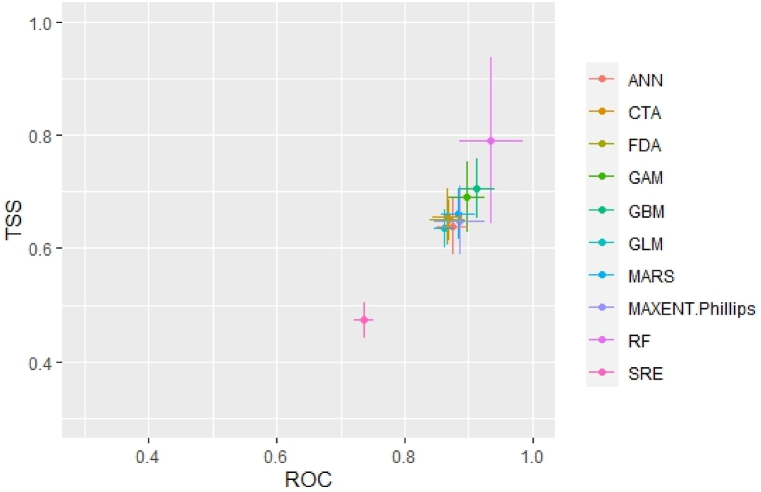
True skill statistics (TSS) and receiver operating characteristic curve (ROC) values of various model methods.

The evaluation of various models utilized metrics such as receiver operating characteristics (ROC) area under the curve (AUC) and true skill statistics (TSS). The weighted mean TSS of ensemble models was 0.741, while AUC values exhibited high performance with a value of 0.943 (Fig. 2). Notably, the RF model demonstrated the highest AUC and TSS values of 0.941 and 0.81, respectively. Statistical accuracy assessments across the ten models consistently identified RF as the top-performing model (Fig. 2). Out of the ten models in Biomod2, models that displayed TSS values greater than 0.625 were considered for the final model running. The lowest accuracy was obtained by the SRE, with AUC and TSS values of 0.730 and 0.470, respectively and hence it was eliminated. Similarly, models CTA, FDA, and GLM were also excluded because of the low AUC values (0.865, 0.870, 0.871, respectively) and current climate suitability maps of these individual models generated are unsatisfactory. Prediction accuracy at the level of each individual algorithm differed with highest AUC for RF (0.941) followed by GBM (0.918), GAM (0.901), MAXENT.Phillips (0.882), MARS (0.881) and ANN (0.878). The corresponding TSS values were RF (0.81) followed by GBM (0.68), GAM (0.68), MARS (0.675), MAXENT.Phillips (0.65) and ANN (0.635).

### 3.2 Bioclimatic variable influence

**Fig. 3:**
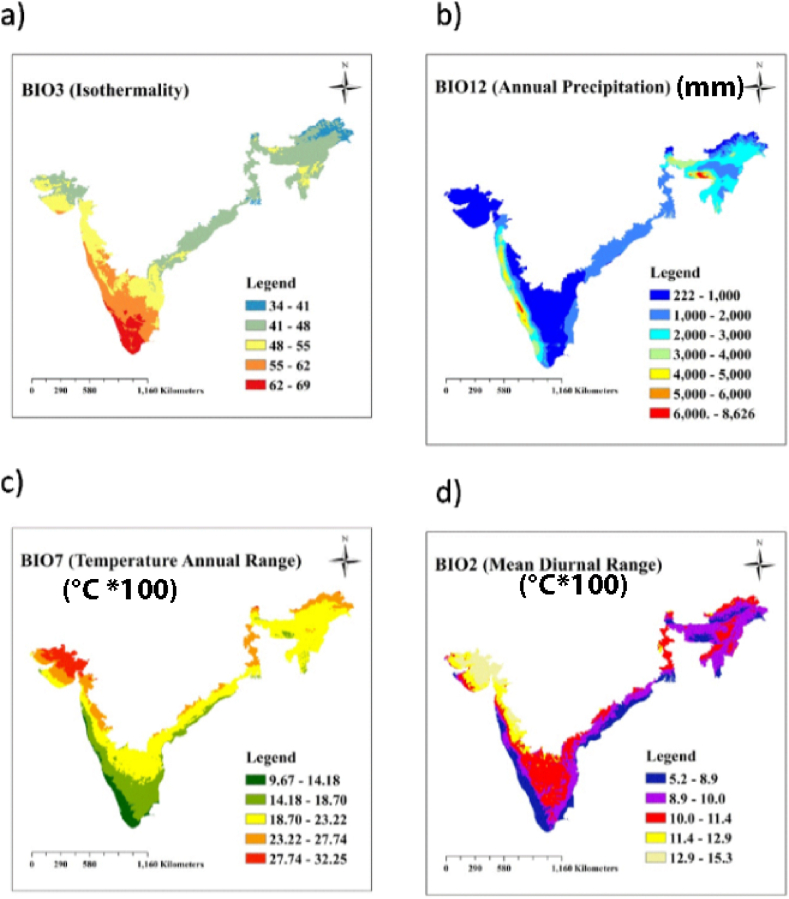
Influence of important Bioclimatic variables on arecanut distribution in study area ie, a) Isothermality (Bio 3), b) Annual Precipitation (mm) (Bio 7), c) Temperature Annual Range (°C *100) (Bio 7) and d) Annual Mean Diurnal Range (°C *100) (Bio 2).

The climatic variables which had high influence in determining the distribution of arecanut are identified and highlighted in Supplementary Table 2. Bio 3 [Isothermality (0.39)] had the highest influence in almost all the algorithms except MARS where it was Bio 7 (annual temperature range). The contribution of Bio 7 and Bio12 (Annual precipitation) was on a par (0.20) followed by Bio 2 (Temperature mean diurnal range, 0.18). The influence of these variables varied widely across the regions of the study area. Diurnal temperature range compared to annual temperature range (Bio 3) (Fig. 3a) and range in temperature extremes (Bio 7) (Fig. 3c) were more variable in east and north east. Meanwhile, the distribution in south interior and some parts of east and north east is determined by annual precipitation (Fig. 3b) and diurnal temperature range (Fig. 3d).

### 3.3 Current and future climate suitability projections

**Fig. 4:**
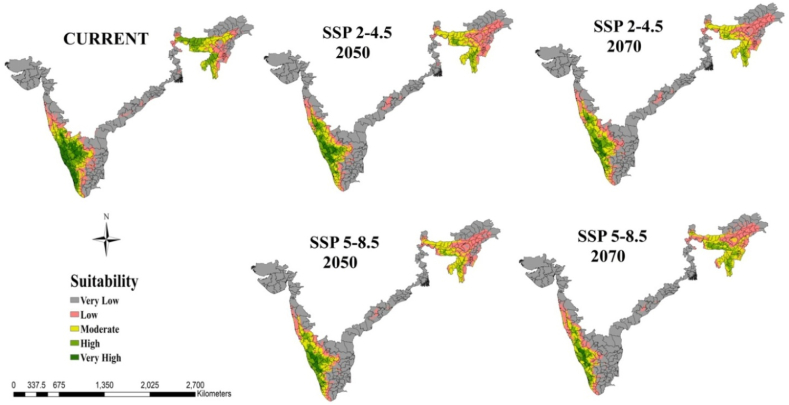
Climatically suitable regions for arecanut production in India for SSP 2–4.5 and SSP 5–8.5 under current and predicted climate in the 2050s and 2070s with ensemble model.

The potential distribution map of arecanut study area projected by ensemble models for current climate and SSP 2–4.5 and 5–8.5 for 2050s and 2070s is presented in Fig. 4 and Table 2. The climates suitable for arecanut cultivation were categorized as Very low (0–200), Low (200–400), Moderate (400–600), High (600–800), and Very High (800–1000) and the analysis of climatic shift from the current to future performed. As per the model, only 69,789 km^2^ or 6% of the study area in India is in the category of ‘Very High’ suited for arecanut production under current environment (Table 2). It mostly occurs in west coast and north east regions (Fig. 4). Climate suitable area is 11% for ‘high’ and ‘moderate’ categories (South interior and north east) while large area is under low (30%) and very low (41%) categories in east. Amongst the future scenarios, SSP 5–8.5 is more severe and most of the very high category will become either moderate or low suitable (73% shift in 2050 and 84% in 2070). The corresponding decline from high category was 37 and 28%, respectively.

**Table 2:** Comparison of arecanut area in India and major arecanut growing states under the current climate and projected climate change during 2050s and 2070s for SSP 2–4.5 and SSP 5–8.5.

Country/state	Category	Current area (Km^2^)	SSP 2–4.5		SSP 5–8.5	
			2050s area (Km^2^)	2070s area (Km^2^)	2050s area (Km^2^)	2070s area (Km^2^)
India (Study Area)	Very Low	465,077	670,933	680,505	701,384	703,119
	Low	341,859	179,324	169,752	179,902	179,859
	Moderate	127,009	155,147	155,147	149,494	143,091
	High	122,533	92,489	92,489	76,428	88,870
	Very High	69,789	28,374	28,374	19,059	11,328
Karnataka	Very Low	1606	16,853	14,819	17,581	44,285
	Low	13,191	19,059	31,757	24,691	31,008
	Moderate	28,845	38,781	40,195	42,807	27,496
	High	51,095	45,099	44,927	43,043	31,672
	Very High	48,139	23,085	11,178	14,754	8416
Kerala	Very Low	664	278	0	43	0
	Low	3084	4154	6296	5503	6467
	Moderate	3876	14,840	13,106	11,414	13,255
	High	8973	13,834	14,583	16,618	15,161
	Very High	20,408	3897	3019	3426	2120
North East	Very Low	117,458	90,733	95,380	100,091	72,359
	Low	52,015	94,459	100,519	103,474	95,744
	Moderate	66,813	83,152	79,597	76,556	86,728
	High	56,063	24,005	16,853	12,228	37,389
	Very High	0	0	0	0	128

### 3.4 Changes in arecanut regional habitat suitability

**Fig. 5:**
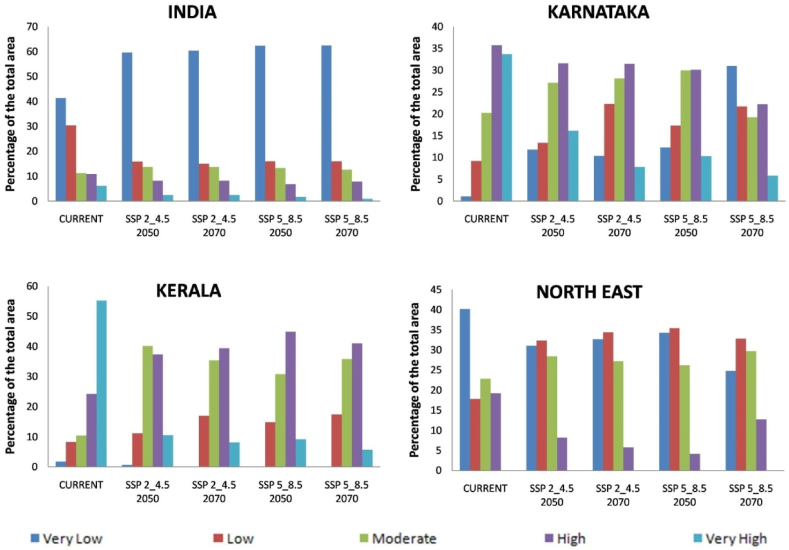
Percentage of total predicted area under different classes for current and future climate of the study area of India and the regional levels of Karnataka, Kerala and North East.

**Fig. 6:**
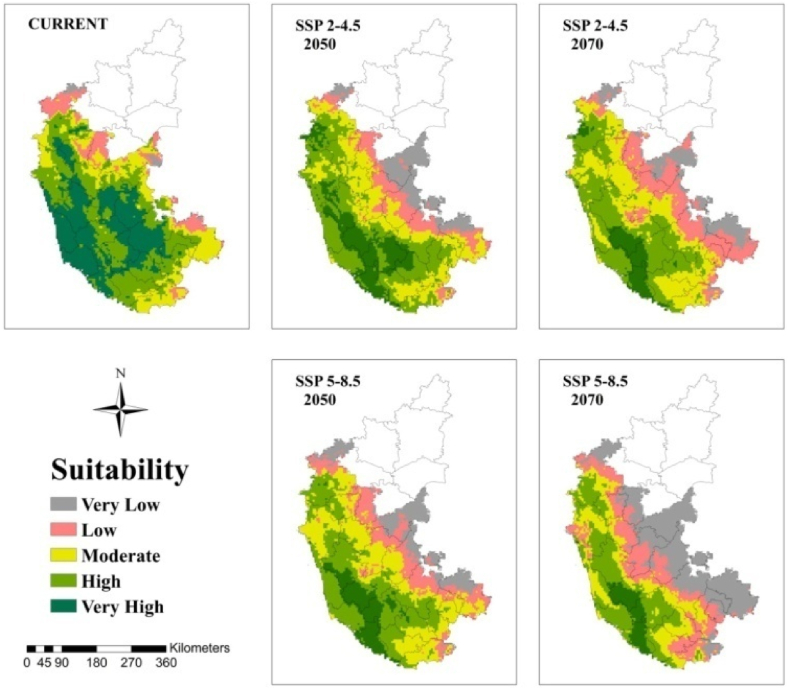
Climatically suitable regions for arecanut production in Karnataka for SSP 2–4.5 and SSP 5–8.5 under present and predicted climate in the 2050s and 2070s.

**Fig. 7:**
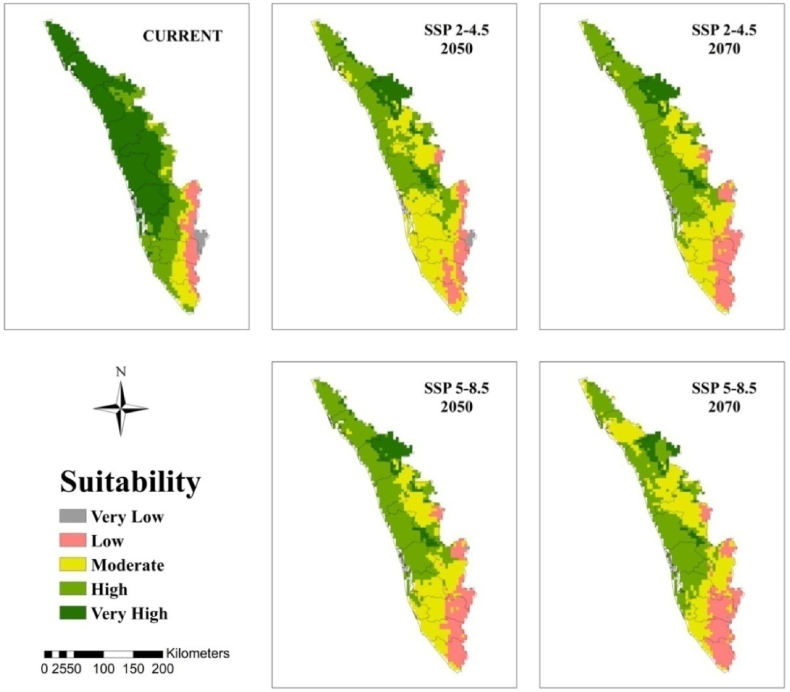
Climatically suitable regions for arecanut production in Kerala for SSP 2–4.5 and SSP 5–8.5 under present and predicted climate in the 2050s and 2070s.

**Fig. 8:**
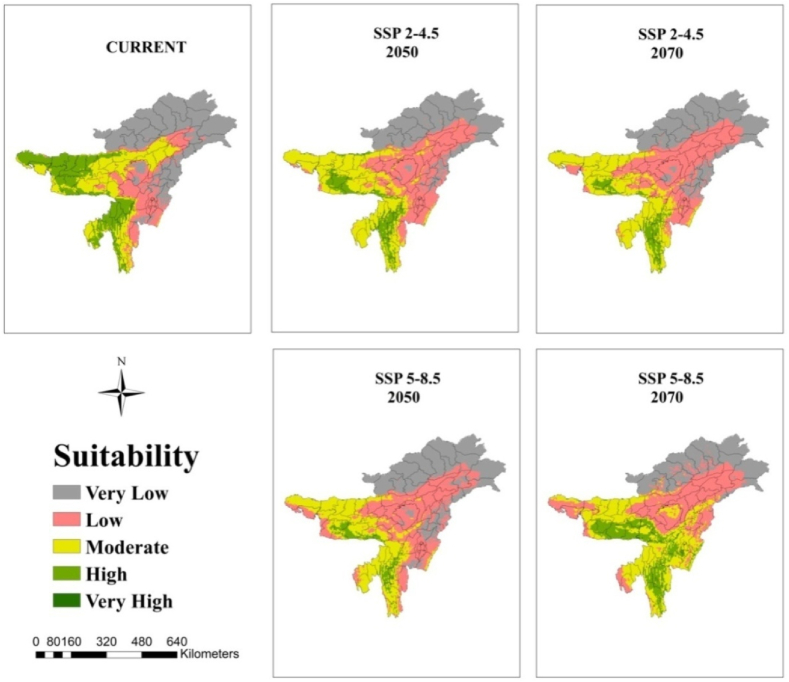
Climatically suitable regions for arecanut production in North Eastern states for SSP 2–4.5 and SSP 5–8.5 under present and predicted climate in the 2050s and 2070s.

At the regional scale, the suitable area for arecanut cultivation in Karnataka and Kerala is roughly 33 and 55% under very high category and 35 and 25% under high category, respectively, under current climate (Fig. 5). In both the states, the area under these categories becomes less than 10% with more severe being under SSP 5–8.5. In Karnataka major shift is towards low to moderate category especially in south interior (Fig. 5, Fig. 6). In Kerala major shift occurs to high and moderate category in north and eastern parts (Fig. 5, Fig. 7). North east India has only 20% under high and 22% area under moderate suitable category under current climate (Fig. 5, Fig. 8). In future scenarios high suitable area shifts to moderate to low category (Table 2). West and south west which is high suitable under current climate mostly becomes moderate suitable under future climate (Fig. 6, Fig. 7, Fig. 8).

## 4 Discussion

This study represents the maiden investigation into the effects of global climate change on the geographical range and environmental habitat suitability of arecanut in India, employing ensemble modeling. Given the arecanut tree's extended lifespan and widespread cultivation across diverse agro-ecological zones in India, the assessment of climate change scenarios on potential cultivable areas proves invaluable. This analysis aids in comprehending the interplay between arecanut niches and their corresponding environments, facilitating the identification of priority cultivation areas and the formulation of strategic adaptation plans [45,46]. Species distribution models (SDMs) like MaxEnt are widely employed to analyze future climate shifts in the plantation growing areas of various crops, including cocoa in African countries [18,47], coffee in Zimbabwe [48], and coconut in India [10]. While the use of a single model for species distribution forecasting is common, there exists a risk of the model either under- or overfitting [49]. Ensemble models, which combine the prediction outcomes of multiple single models, serve to mitigate uncertainty associated with model fitting [40]. Consequently, they enhance prediction accuracy, provide better generality, and exhibit greater robustness compared to individual models [50]. The “biomod2” package that serves as an object-oriented, extensible, and reproducible R platform for implementing these ensemble models [25,51] was used to predict the future climate suitable regions for arecanut. The ensemble model had ten model algorithms, however in this study we used six models (GAM, GBM, ANN, RF, MaxEnt and MARS) to create the final output because of their high TSS values (more than 0.625) and accuracy. Typically, TSS scores exceeding 0.6 are deemed satisfactory [39,42]. The findings indicate that the ensemble model's forecast for arecanut yields mean AUC values of 0.943 and TSS values of 0.741, placing it in the excellent category, aligning with prior research [52,53]. Within our investigations, the RF algorithm achieved the highest TSS value (0.81), while ANN recorded the lowest (0.63), with an average TSS value of 0.741 across the six models.

The ensemble model indicated that the future cultivation of arecanut is primarily influenced by bioclimatic variables, with temperature isothermality (Bio 3) playing the most significant role in determining habitat suitability. Additionally, temperature range (Bio 7) and annual precipitation (Bio 12) also contributed significantly to the suitability of the habitat.

Earlier reports suggest that arecanut depends on, relative humidity and precipitation [5,6]. Climatic factors such as T_max_, T_min_, rainfall and RH could determine more than 97% variation in yield of areca nut [7]. There was significant positive correlations between arecanut yield and T_max_ (r = 0.48), T_min_ (r = 0.16) and RH (r = 0.32 to 0.49) [9]. It grows reasonably well at a temperature of 16–30 °C but beyond 36 °C the growth of the crop is significantly affected [3]. In India, T_max_ of some of the arecanut growing regions is >36 °C eg., south interior Karnataka (eg., districts of Chitradurga, Tumkur, Shivamogga, Table 1) while in north east the temperature during winter is well below 16 °C (Assam). The deleterious effects of high or low temperatures are not well studied in arecanut. Nevertheless, elevated temperatures negatively impact photosynthesis in coconut seedlings [54]. During the reproductive phase, high temperatures hinder pollen germination [55], limit pollen tube expansion through the style [8], ultimately leading to reduced fertilization and nut set. Consequently, palms are unable to withstand temperature extremes and significant diurnal variations. Ensemble model prediction of climatic variables is in conformity with the above observations; arecanut in south interior regions are to suffer from low precipitation (Bio12) and wider diurnal temperature range (Bio7), whereas in east it is high temperature and north east it is wide variability of temperature across the seasons (Bio 3). In India west coast is a high rainfall area with T_max_ around 36 °C is ideal for arecanut cultivation.

Arecanut is a perennial plant with a long life span of 45–60 years. Therefore before planting it is pertinent to know the current and future climate of the planting region. For instance, during 1950s the western foot hills of Western Ghats of Kerala and Karnataka were the traditional belts of arecanut cultivation. During the last 70 years, arecanut in these regions, especially in Kerala, was almost wiped out due the prevalence of a malady called yellow leaf disease [27] believed to be caused by phytoplasma [56]. However, during the same period its cultivation has rampantly spread in non-traditional areas of Karnataka (nearly 10 folds increase in area) and the spread is very high in recent years. Better economic return of arecanut than other crops of the region is the major driving force for its rampant cultivation. Though arecanut is fetching a high price now, this unchecked spread of arecanut has worried policy makers and others if it could become vulnerable to climatic variables in future. In that context it is important to identify climatically suitable and vulnerable areas for new plantations of arecanut and to suggest adaptive strategies in those areas where it is planted and is becoming vulnerable.

Application of Ensemble model under current climate scenario had projected that all along the western foot hills of Western Ghats (mostly in Karnataka and Kerala) is very high (6% of total area) suitable for arecanut cultivation. Rest of the west and part of north east was observed to be high suitable (11%) while rest of north east and south interior the projection was moderate suitable (11%). This projection is in conformity with the current cultivation area as shown in Table 2.

Our previous investigation in coconut, along with other documented reports, has illustrated that the climate of the region, influenced by global warming, has led to expansions, shifts, or contractions in the cultivated area [10,12]. From Fig. 5 and Table 2 it is apparent that area expansion is observed which is quite small under ‘moderate’ category while major area expansion is happening in ‘very low’ category suggesting more area is becoming vulnerable for arecanut cultivation. This shift is mostly coming from ‘very high’ and ‘high’ category. Scenario SSP5-8.5 reveal a shift in area from ‘very high’ and ‘high’ categories to ‘moderate’ and ‘very low’ categories compared to SSP2-4.5. Karnataka, where rapid area expansion of arecanut is happening, currently has 0.5 m ha of the total 0.73 m ha of India (http://dasd.gov.in), is highly vulnerable and large area of east of Karnataka is shifting from ‘high’ and ‘moderate’ to ‘moderate’, ‘low’ and ‘very low’ category regions. Similarly in Kerala, northern and eastern parts are becoming less suitable. Currently, the north west in the north east is highly suitable, but it is expected to shift to the south. Such shifts, expansions, and contractions in a specific region due to climate variables are commonplace and align with previous studies suggesting potential replacements, such as the shift from coffee to cocoa in Mesoamerica [57], cocoa in Latin America [58], cocoa in West Africa [47], coffee in Zimbabwe [59], and coconut in India [10].

Regions categorized as ‘low’ and ‘very low’ vulnerability (East of Karnataka, North and east of Kerala) face heightened susceptibility to climate scenarios. Consequently, it is strongly recommended to refrain from initiating new areca plantations in these areas. Conversely, special attention and additional protective measures should be directed towards arecanut cultivation in the south interior regions, specifically in Chitradurga, Tumkur, and Shivamogga districts of Karnataka. This strategic approach aims to ensure the sustainable cultivation of arecanut, particularly in existing cultivated areas. In these identified regions, factors such as low precipitation, high temperatures, and low humidity significantly contribute to shifting suitability from ‘very high’ and ‘high’ to ‘moderate.’ The impact of elevated temperatures can be mitigated to some extent by introducing genotypes with broader adaptability to high temperatures and water deficits. Alternatively, the adoption of agricultural techniques like fertigation [60], coupled with soil moisture conservation practices such as mulching, bunding, and cropping systems [61], can prove beneficial. Implementing strategies like soil moisture conservation, summer irrigation, and drip fertigation represents key agronomic adaptations. These measures not only serve to minimize losses but also enhance productivity across a majority of arecanut-growing areas. Ensuring water availability to the palms could aid in canopy cooling through transpiration, partially offsetting the adverse effects of high temperatures [62]. In the north east, where the temperature is low during winter and wide variability between the seasons exists, the genotypes that exhibit tolerance to both low and high temperatures are likely to provide resilience to the crop in the face of future climate conditions. Adoption of agronomic measures suitable for the region will be a key factor in overcoming the ill effects of climate change apart from suitable genotypes.

## 5 Conclusions

Arecanut, an important plantation crop is grown in foot hills and plains of south and north east India. As it has long life span and grown in different agroecological zones it is exposed to varied climatic variables. Ensemble model, Biomod2, used in this study had quite accurately predicted the current regions of climate suitability and likely shift, expansion and contraction of climate suitable areas under two future scenarios (SSP 2–4.5 and SSP 5–8.5 of 2050 and 2070). Model had predicted that in south interior of Karnataka, which is the major arecanut growing belt, cultivation will be limited by amount of precipitation and temperature range while in north east the diurnal temperature across the seasons is a major limiting factor. Parts of eastern Karnataka and north Kerala are likely to become very low suitable and advised not to establish new arecanut plantations. While majority of the present ‘very high’ and ‘high’ suitable regions are becoming ‘moderately’ suitable, there is a need to plant tolerant varieties and adopt agro-techniques to sustain the current level of production. However, in arecanut the response of the crop to adverse climatic factors (high temperature, drought etc.) is poorly understood. Therefore, appropriate phenotyping and genotyping of arecanut to abiotic factors is an important research prerequisite to device proper adaptive measures.

## Funding

This work was supported by funding from ICAR-Central Plantation Crops Research Institute (ICAR-CPCRI Grant No: 1000766014).

## Data availability statement

All data generated or analyzed during this study are included in this published article.

## CRediT authorship contribution statement

**K.B. Hebbar:** Writing – review & editing, Writing – original draft, Supervision, Resources, Project administration, Investigation, Funding acquisition, Formal analysis, Conceptualization. **Abhin Sukumar P:** Investigation, Formal analysis. **Sanjo Jose V:** Resources, Investigation, Formal analysis. **Ramesh S V:** Writing – review & editing, Writing – original draft, Formal analysis. **Ravi Bhat:** Writing – review & editing, Formal analysis.

## Declaration of competing interest

The authors declare that they have no known competing financial interests or personal relationships that could have appeared to influence the work reported in this paper.
