# Supplementary material for: Predicting current and future climate suitability for arecanut (Areca catechu L.) in India using ensemble model
Source: Heliyon. 2024 Feb 16;10(4):e26382. doi: 10.1016/j.heliyon.2024.e26382 (PMC10901027; doi:10.1016/j.heliyon.2024.e26382)
Supplement: Multimedia component 1 [file mmc1.docx]

**Supplementary files**

Supplementary Table 1: Pearson correlation among the nineteen different bioclimatic variables used in modeling

|  | **Bio 1** | **Bio 2** | **Bio 3** | **Bio 4** | **Bio 5** | **Bio 6** | **Bio 7** | **Bio 8** | **Bio 9** | **Bio 10** | **Bio 11** | **Bio 12** | **Bio 13** | **Bio 14** | **Bio 15** | **Bio 16** | **Bio 17** | **Bio 18** | **Bio 19** |
| --- | --- | --- | --- | --- | --- | --- | --- | --- | --- | --- | --- | --- | --- | --- | --- | --- | --- | --- | --- |
| **Bio 1** | 1 |  |  |  |  |  |  |  |  |  |  |  |  |  |  |  |  |  |  |
| **Bio 2** | 0.14 | 1.00 |  |  |  |  |  |  |  |  |  |  |  |  |  |  |  |  |  |
| **Bio 3** | 0.41 | -0.06 | 1.00 |  |  |  |  |  |  |  |  |  |  |  |  |  |  |  |  |
| **Bio 4** | -0.50 | 0.28 | **-0.88** | 1.00 |  |  |  |  |  |  |  |  |  |  |  |  |  |  |  |
| **Bio 5** | **0.94** | 0.42 | 0.29 | -0.34 | 1.00 |  |  |  |  |  |  |  |  |  |  |  |  |  |  |
| **Bio 6** | **0.90** | -0.15 | 0.66 | -0.79 | 0.76 | 1.00 |  |  |  |  |  |  |  |  |  |  |  |  |  |
| **Bio 7** | -0.17 | 0.79 | -0.65 | 0.78 | 0.11 | -0.55 | 1.00 |  |  |  |  |  |  |  |  |  |  |  |  |
| **Bio 8** | **0.90** | 0.18 | 0.07 | -0.13 | **0.83** | 0.66 | 0.07 | 1.00 |  |  |  |  |  |  |  |  |  |  |  |
| **Bio 9** | **0.94** | 0.14 | 0.61 | -0.68 | **0.88** | **0.94** | -0.30 | 0.72 | 1.00 |  |  |  |  |  |  |  |  |  |  |
| **Bio 10** | **0.98** | 0.26 | 0.26 | -0.33 | **0.97** | **0.81** | 0.01 | **0.93** | **0.89** | 1.00 |  |  |  |  |  |  |  |  |  |
| **Bio 11** | **0.96** | 0.04 | 0.61 | -0.72 | **0.87** | **0.97** | -0.37 | 0.76 | **0.97** | **0.90** | 1.00 |  |  |  |  |  |  |  |  |
| **Bio 12** | -0.14 | -0.53 | -0.03 | -0.11 | -0.34 | -0.04 | -0.38 | -0.06 | -0.18 | -0.21 | -0.09 | 1.00 |  |  |  |  |  |  |  |
| **Bio 13** | -0.04 | -0.36 | 0.11 | -0.24 | -0.19 | 0.06 | -0.33 | -0.05 | -0.02 | -0.12 | 0.04 | **0.92** | 1.00 |  |  |  |  |  |  |
| **Bio 14** | -0.18 | -0.46 | 0.07 | 0.03 | -0.35 | -0.07 | -0.34 | -0.12 | -0.22 | -0.23 | -0.18 | 0.21 | -0.03 | 1.00 |  |  |  |  |  |
| **Bio 15** | 0.22 | 0.47 | -0.05 | 0.04 | 0.32 | 0.01 | 0.39 | 0.24 | 0.23 | 0.26 | 0.17 | 0.08 | 0.37 | -0.57 | 1.00 |  |  |  |  |
| **Bio 16** | -0.08 | -0.42 | 0.03 | -0.17 | -0.25 | 0.00 | -0.33 | -0.05 | -0.09 | -0.15 | -0.02 | **0.96** | **0.99** | 0.04 | 0.29 | 1.00 |  |  |  |
| **Bio 17** | -0.20 | -0.53 | -0.01 | 0.08 | -0.39 | -0.10 | -0.35 | -0.09 | -0.28 | -0.25 | -0.21 | 0.32 | 0.03 | **0.94** | -0.59 | 0.12 | 1.00 |  |  |
| **Bio 18** | -0.36 | -0.35 | -0.40 | 0.39 | -0.50 | -0.40 | -0.02 | -0.10 | -0.49 | -0.36 | -0.43 | 0.67 | 0.41 | 0.35 | -0.20 | 0.51 | 0.46 | 1.00 |  |
| **Bio 19** | 0.09 | -0.29 | 0.37 | -0.35 | -0.02 | 0.25 | -0.41 | -0.04 | 0.17 | 0.02 | 0.18 | 0.40 | 0.44 | 0.05 | 0.04 | 0.44 | 0.08 | -0.07 | 1 |

Supplementary Table 2. Bioclimatic factors selected for modeling the climatic suitability of arecanut

|  | **GBM** | **GAM** | **ANN** | **MARS** | **RF** | **MAXENT** | **Mean** |
| --- | --- | --- | --- | --- | --- | --- | --- |
| **bio_1** | 0.10 | 0.20 | 0.08 | 0.27 | 0.11 | 0.14 | 0.15 |
| **bio_2** | 0.01 | 0.66 | 0.08 | 0.24 | 0.04 | 0.07 | 0.18 |
| **bio_3** | 0.38 | 0.81 | 0.43 | 0.29 | 0.20 | 0.23 | 0.39 |
| **bio_7** | 0.08 | 0.42 | 0.16 | 0.36 | 0.12 | 0.08 | 0.20 |
| **bio_12** | 0.03 | 0.42 | 0.42 | 0.16 | 0.06 | 0.10 | 0.20 |
| **bio_14** | 0.05 | 0.17 | 0.23 | 0.15 | 0.07 | 0.07 | 0.13 |
| **bio_15** | 0.02 | 0.27 | 0.23 | 0.10 | 0.05 | 0.04 | 0.12 |
| **bio_18** | 0.08 | 0.04 | 0.43 | 0.08 | 0.07 | 0.05 | 0.12 |
| **bio_19** | 0.04 | 0.12 | 0.34 | 0.11 | 0.05 | 0.18 | 0.14 |
